# Light therapy for seasonal affective disorder: correspondence

**DOI:** 10.1097/MS9.0000000000000166

**Published:** 2023-01-18

**Authors:** Hitesh Chopra, Muhammad S. Khan, Simona Cavalu, Pradipta R. Rauta, Kuldeep Dhama, Talha B. Emran

**Affiliations:** a Chitkara College of Pharmacy, Chitkara University, Rajpura, Punjab; b Department of Biological Sciences, AIPH University, Bhubaneswar, Odisha; c Division of Pathology, ICAR-Indian Veterinary Research Institute, Bareilly, Izatnagar, Uttar Pradesh, India; d Department of Biosciences, Faculty of Sciences, COMSATS University Islamabad, Sahiwal, Pakistan; e Faculty of Medicine and Pharmacy, University of Oradea, Oradea, Romania; f Department of Pharmacy, BGC Trust University Bangladesh, Chittagong, Bangladesh; g Department of Pharmacy, Faculty of Allied Health Sciences, Daffodil International University, Dhaka, Bangladesh

*Dear Editor*,

For brief periods of time, many individuals experience feelings of sadness or a change in their normal disposition. Mood swings might be temporary, starting, and stopping with the seasons. When the days become shorter in the autumn and winter, some people experience a feeling of depression known as ‘winter blues’, which often lifts when spring arrives and longer hours of sunshine return. Mood swings may have a profound effect on a person’s emotions, thoughts, and ability to function in everyday life. Seasonal affective disorder (SAD) is a kind of sadness characterized by major changes in mood and behavior that occur with each new season.

The most common form of SAD, also known as ‘winter-pattern SAD’ or ‘winter depression’, is characterized by the onset of symptoms in late autumn or early winter and their subsequent resolution in the spring and summer. Instead of being its own disease, SAD is just a subtype of sadness that occurs every year at the same time. Thus, the symptoms of SAD include those of severe depression as well as those unique to either SAD in the winter or SAD in the summer. The exact origins of SAD are still a mystery to researchers. The neurotransmitter serotonin plays an important role in mood regulation, and studies suggest that people with SAD may have lower levels of this molecule. According to studies, patients with SAD have impaired regulation of the amounts of molecules that assist maintain normal serotonin levels, leading to low levels of serotonin in the winter^1^. One theory for why SAD sufferers cannot keep their sleep–wake cycles in check is that they create too much of the hormone melatonin. An overabundance of melatonin might make you feel sleepy. The seasonal night–day cycle is reflected in the body’s daily rhythm, which is maintained in part by the hormone’s serotonin and melatonin. Alterations in serotonin and melatonin levels are associated with SAD, which causes sufferers to experience disruptions in their daily cycles. Because of this, they experience shifts in sleep, mood, and behavior according to the seasonal variations in day duration.

The sleep–wake cycle and the endogenous circadian rhythm are best when they are in sync, according to the ‘phase-shift hypothesis’, the current leading theory for the etiology of SAD. As the days become shorter in the autumn and winter, the natural body clock (circadian rhythm) starts to advance in relation to the time of day and the sleep–wake cycle. There is speculation that this phase difference contributes to emotional distress.

Clues to the pathophysiology of SAD were initially disclosed in the inaugural research by Rosenthal *et al*.^2^ which found that exposing individuals with winter SAD to strong white light, thus increasing their photoperiod (day exposure to light), improved their depressive symptoms. While full-spectrum fluorescent light is used in most light boxes and other light therapy (LT) equipment, other colors of light, such as red, blue, and green, may also have therapeutic effects. At least 30 minutes per day should be spent exposed to artificial light (through a light box or visor) in order to get this effect. Only under medical supervision and with US Food and Drug Administration-approved equipment may LT be utilized. Tanning beds and other artificial sources of ultraviolet radiation pose health risks.

A dawn simulator is a time-controlled light that is used to simulate the sunrise as a therapy for helping to reset the body’s internal clock. The lux is the standard unit for evaluating a SAD lamp’s output. The unit of measurement for light intensity and surface area is the lux. It is recommended that a SAD lamp provide 10,000 lux of illumination. To prevent eye strain from glare, a lamp’s viewing angle should enable it to be held slightly above the eyes and at an inclination downward lamps and lights designed to treat SAD should be used for 30 minutes to an hour daily. You may use them whenever you choose, but they work best first thing in the morning.

It has been hypothesized that LT may function by either correcting the winter circadian rhythm phase delay or by increasing synaptic serotonin, possibly in the serotonin-rich midbrain, a target of retinofugal pathways, or perhaps by both mechanisms, based on the proposed pathophysiologies of SAD described above. LT applied to the popliteal fossa in the knee of individuals with SAD was demonstrated to have no impact on symptoms, suggesting that the therapeutic benefits of LT seem to need the eyes (and light-activated retinofugal pathways)^3^. In addition to melanopsin and opsin, additional light-absorbing molecules, including hemoglobin, biliverdin, and bilirubin, and gaseous molecules, such as CO and NO, found in retinal blood vessels, have been speculated to have a role in the mechanism of action of LT in recent research^4^.

In addition to more strong randomized controlled trials for the use of LT in illnesses other than SAD, greater study into the physiological processes of LT is required. Such studies would help us better understand the pathophysiological processes of the illnesses impacted by LT and the pathways subserving the beneficial effects of LT, as well as the ideal circumstances for light treatment for each ailment. Regardless, LT has a very promising future in psychiatry, both as an adjuvant and as a standalone treatment.

## Ethical approval

This case report has been reported in line with the Case Report (CARE) guidelines.

## Consent

Not applicable.

## Source of funding

This study received no specific grant from any funding agency in the public, commercial, or not-for-profit sectors.

## Authors’ contribution

H.C.: conceptualization, data curation, writing – original draft preparation, writing – reviewing and editing. M.S.K., S.C., P.R.R., K.D.: data curation, writing – original draft preparation, writing – reviewing and editing. T.B.E.: writing – reviewing and editing, visualization, supervision. All the authors confirm the concept, design, data collection and analysis and interpretation, and writing are our own.

## Conflict of interest disclosure

We have read and understood the policy on declaration of interests and have no relevant interests to declare. The responsibility for the content lies with the author and the views stated herein should not be taken to represent those of any organizations or groups with and for which he works.

## Research registration number (UIN)

None.

## Guarantor

Talha Bin Emran, PhD, Associate Professor, Department of Pharmacy, BGC Trust University Bangladesh, Chittagong 4381, Bangladesh. Tel: +880 303 356 193, Fax: +880 312 550 224. https://orcid.org/0000-0003-3188-2272. E-mail: talhabmb@bgctub.ac.bd

## Provenance and peer review

Not commissioned, externally peer reviewed.
